# HoxA Genes and the Fin-to-Limb Transition in Vertebrates

**DOI:** 10.3390/jdb4010010

**Published:** 2016-02-17

**Authors:** João Leite-Castro, Vanessa Beviano, Pedro Nuno Rodrigues, Renata Freitas

**Affiliations:** 1 IBMC—Instituto de Biologia Celular e Molecular, Oporto, Portugal; newtonjplc@hotmail.com (J.L.-C.); vanessa.beviano@hotmail.com (V.B.); PRodrigu@ibmc.up.pt (P.N.R.); 2 I3S—Instituto de Investigação e Inovação em Saúde, Rua Alfredo Allen, 208, 4200-135 Porto, Portugal; 3 ICBAS—Instituto de Ciências Biomédicas Abel Salazar, University of Oporto, Rua de Jorge Viterbo Ferreira n°. 228, 4050-313 Oporto, Portugal

**Keywords:** *HoxA* genes, transcriptional regulation, development, evolution, limb, fin

## Abstract

*HoxA* genes encode for important DNA-binding transcription factors that act during limb development, regulating primarily gene expression and, consequently, morphogenesis and skeletal differentiation. Within these genes, *HoxA11* and *HoxA13* were proposed to have played an essential role in the enigmatic evolutionary transition from fish fins to tetrapod limbs. Indeed, comparative gene expression analyses led to the suggestion that changes in their regulation might have been essential for the diversification of vertebrates’ appendages. In this review, we highlight three potential modifications in the regulation and function of these genes that may have boosted appendage evolution: (1) the expansion of polyalanine repeats in the HoxA11 and HoxA13 proteins; (2) the origin of +a novel long-non-coding RNA with a possible inhibitory function on *HoxA11*; and (3) the acquisition of *cis*-regulatory elements modulating 5’ *HoxA* transcription. We discuss the relevance of these mechanisms for appendage diversification reviewing the current state of the art and performing additional comparative analyses to characterize, in a phylogenetic framework, *HoxA11* and *HoxA13* expression, alanine composition within the encoded proteins, long-non-coding RNAs and *cis*-regulatory elements.

## 1. Introduction

The autopod, the multi-fingered extremity located at the end of a hindlimb or forelimb, is a specific characteristic of tetrapods [1,2,3]. The fossil record suggests that this anatomical structure evolved from sarcopterygian fins by sequential expansion and elaboration of their endochondral bones and concomitant reduction of the apical dermoskeleton [1,2,3]. Regarding the number of digits, both paleontological and developmental data suggest an evolutionary sequence that went from an adactyl state detectable up to the divergence of sarcopterygians [4], then sidestepping by digit-like radials in stem sarcopterygians [5], polydactyly in stem tetrapod and culminating in the fixation of pentadactyl [6,7,8]. From a developmental point of view, understanding the molecular mechanisms that enabled the fin-to-limb transition is a puzzling question that has long held the attention of the scientific community and holds the potential of enlightening molecular biologists on how gene regulatory networks and interactions evolved.

The patterning of the skeletal elements, in both fin and limb, largely relies on the action of signaling centers operating from the onset of their outgrowth: the apical ectodermal ridge (AER) and the zone of polarizing activity (ZPA) [9]. However, there are marked differences in the morphology and development of the most distal part of fins and limbs. In tetrapods, the influence of the AER is maintained throughout limb development, being important for the differentiation of the stylopod, zeugopod and autopod [10,11,12]. In fish, however, this structure is quickly converted into a finfold (FF) during fin development that then gives rise to the apical dermoskeleton [13]. However, its function seems to be conserved during fish fin development and relates to the coordination of the proximo-distal patterning [14]. Recent studies performed in zebrafish suggest that the timing of the AER-FF transition may have mediated the differences found between fins and limbs and that the acquisition of a mechanism repressing the formation of the FF may have been crucial in the development of tetrapod limbs [15].

The patterning of the endoskeleton structure has been proven to rely on the expression of 5’ *HoxA* and *HoxD* genes in both fins and limbs, as demonstrated by different studies performed on a wide variety of model systems [16,17,18,19,20]. Indeed, several lines of evidence highlight the role of these transcriptional factors as key intervenients in limb development. For example, loss-of-function mutations in genes located at the 5’ end of the mouse HoxA and HoxD clusters lead to the appearance of limbs with atavistic characters [8,18,20]. The impact that these genes have on the patterning of the limb, proven by loss-of-function assays, led Duboule and colleagues to suggest that modifications on Hox regulation may have been a source of morphological variation during the evolution of tetrapod limbs [21]. Moreover, they suggest that increased levels of these transcription factors during fin development may have caused extra-proliferation distally, setting the grounds for the formation of additional endoskeleton elements, which occurred concomitantly with the reduction of the apical dermoskeleton. To make the proof of principle of these ideas, Freitas and colleagues overexpressed the zebrafish counterpart of *HoxD13* (*hoxd13a*), generating a fin phenotype characterized by additional endochondral tissue and reduction or ablation of the apical finfold [22]. Thus, *hoxd13a* overexpression induces a phenotype that mirrors two of the major morphological alterations marked as crucial in limb evolution by the fossil record: endochondral expansion and dermoskeleton retraction [1,22]. 

Taking into account the results obtained by Zákány *et al.* [8] and Freitas *et al.* [22], it seems that both HoxA and HoxD clusters contributed to the fin-to-limb transition, although at distinct time points and by regulating different cellular events. Zákány and colleagues analyzed the phenotypes of several compound mouse mutants with distal *HoxA* and *HoxD* loss-of-function alleles and proposed that the *HoxA13* gene was the initial/primordial major contributor for the determination of the autopod identity [8]. This was probably followed, in the course of evolution, by the recruitment of distal *HoxD* genes (*HoxD11*, *HoxD12* and *HoxD13*), whose expression is associated with finger elongation and fixation of [8,23]. Further evidences that supports this sequence of events comes from the observation that *HoxA13* is expressed earlier than *HoxD13* in the distal mesenchyme during limb development [23], being probably an earlier specifier of the autopod identity. Moreover, the expression of the *Hox13* paralogs in zebrafish is detected in the most distal mesenchyme of the developing fins, closely mirroring the expression patterns described in tetrapods [17]. This suggests that *HoxA13* was already essential to induce a distal appendage identity even prior to the origin of the autopod in the tetrapod lineage. Taken together, these data suggest that alterations in the expression and function of HoxA13 may have been the primary mechanisms leading to the formation of the autopod in the tetrapod lineage [24,25]. 

Taking into account the conserved *hoxa13* expression patterns found distally in developing fish fins, why do they not give rise to autopod-like structures? Information gathered from developmental, phylogenetic and gene function studies suggests that, in the course of evolution, two factors had to meet to allow the formation of an autopod domain, those being the alteration of the proximo-distal patterning leading to the formation of novel distal territories, characterized by a specific transcriptome, that co-occurred with, or was followed by, an increase in the levels of *HoxA13* transcripts [8,26]. 

**Figure 1:**
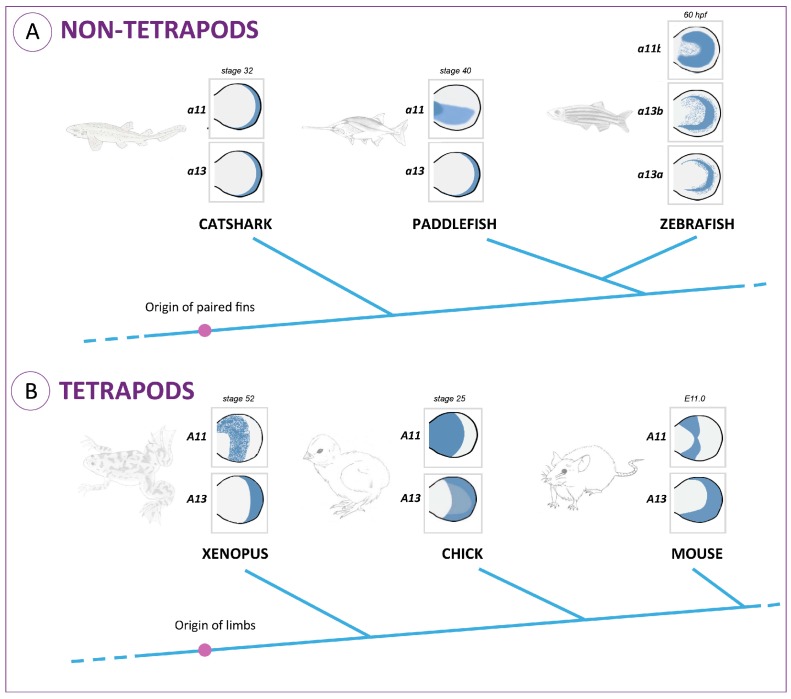
Expression patterns of 5’ *HoxA* genes during the development of paired appendages in vertebrates. The selection of stages took into account data from: *Xenopus* [16]; chick [23]; mouse [31]; catshark [35]; paddlefish [36]; zebrafish [17]. (**A**) Expression domains of *HoxA11* and *HoxA13* in developing fins from fish representing phylogenetic groups that diverged prior to the origin of tetrapods; (**B**) Expression domains of *HoxA11* and *HoxA13* in developing limbs in representatives of tetrapod lineages (in blue).

Regarding the proximo-distal patterning, striking differences have been found in the expression of 5’ *HoxA* genes in fish and tetrapod models that may underlie an increased endoskeletal complexity throughout fin/limb evolution (Figure 1). This process counts with the combined action of three genes encoding master regulators of transcription: *Meis1*, which seems to be essential for stylopod specification; *HoxA11* required for proper zeugopod development; and *HoxA13*, which contributes to an autopod identity [27,28,29,30,31]. Interestingly, during tetrapod limb development, the initially overlapping expression domains of *HoxA11* and *HoxA13* split up to a point in which *HoxA11* is exclusively expressed in the prospective zeugopod, while *HoxA13* is restricted to the future autopod (Figure 1) [16,32,33]. The expression patterns of these genes during fish fin development are, however, quite different. In zebrafish, a full separation of the *hoxa11* and *hoxa13* expression domains has never been reported in the developing fins [17,21,34]. In other fish representing distinct phylogenetic positions, such as chondrichthyans (catshark; [35]) and basal actinopterygians (paddlefish; [26,36]), there is only a transient separation of *hoxa11* and *hoxa13* expression domains during early stages of fin development. These data suggest that the decoupling of *HoxA13/HoxA11* expression domains may have occurred in the common ancestor of gnathostomes and was secondarily lost in a particular lineage of actinopterygians, the teleosts.

The origin of a distal domain exclusively expressing *HoxA13* may have influenced the formation of novel endoskeleton structures [26,37,38]. Further support of this hypothesis is provided by regeneration studies in *Xenopus*, where non-separation of *HoxA11* and *HoxA13* expression patterns, making distal blastemal cells *HoxA11* and *HoxA13* double positive, appeared to be associated with the inability to regenerate an autopod [16]. Interestingly, *HoxA11* and *HoxA13* mRNA are co-expressed in the developing limbs of axolotls, but the translated HoxA11 and HoxA13 proteins are distributed in distinct domains consistent with the formation of a zeugopod-autopod border [39]. Questions that remain to be answered are, for example: How was the separation of HoxA11 and HoxA13 domains achieved, and what were the implications for the evolution of the limb morphology? What were the molecular mechanisms associated with this alterations? Or, how did alterations in HoxA11 and HoxA13 protein levels contribute to the autopod origin? 

From a gene regulatory perspective, there are several mechanisms that could have contributed to the establishment of a *HoxA13* single positive distal appendage domain that allowed the formation and posterior evolution of the autopod. These may include: (1) alteration in the signaling pathways that act upstream of *HoxA13*, regulating its expression and/or the expression of *HoxA11*; (2) remodeling/alteration or elaboration of the *cis*-regulatory motifs that assume the spatial-temporal control of the expression for these genes; (3) acquisition/loss of elements of the post-transcriptional gene regulatory network; (4) alteration of the protein-protein or protein-DNA interactions through alteration of the coding sequence. 

By analyzing the reports published to date, there are three mechanisms that may uphold an evolutionary relevance in the context of the fin-to-limb transition, those being the acquisition/expansion of *HoxA11* and *HoxA13* homopolymeric repeats, acquisition of a new long-non-coding RNA with possible *HoxA11* inhibitory function and the origin of novel *cis*-regulatory regions acting on *HoxA11* and *HoxA13* [40,41,42,43]. Here, we will explore the evolutionary/developmental relevance of these three mechanisms, reviewing the current state of the art concerning *HoxA11* and *HoxA13* regulation throughout development in species at different phylogenetic positions and discussing how these developmental regulatory networks may have evolved, being at the core of the morphological diversification found in vertebrate appendages.

## 2. Polyalanine Repeats and the Fin-to-Limb Transition

Amino acid repeats are common among eukaryote proteins, and their length and frequency cannot be explained solely by random chance, suggesting a biological function and positive selection [44,45]. Interestingly, prokaryote proteins are impoverished in amino acid repeats, when compared to their eukaryotic homologues, indicating that evolution of such amino acid repeats happened after establishment of the initial protein structures and possibly concomitantly with the appearance of novel functionalities in eukaryotes [44]. Indeed, numerous proteins with a variety of different biological functions display amino acid repeats, such as protein kinases, signaling proteins, membrane transporters and transcription factors [44]. Strikingly, the majority of amino acid repeats detected in eukaryotes are present in proteins involved in the formation of large protein-protein or protein-nucleic acid complexes, such as transcriptional factors, possibly influencing their performance [44]. Thus, changes in these particular sequences during evolution may have produced a significant impact, leading to the genesis of novel morphological structures, for example. 

Interestingly, alterations in the length of amino acid tandem repeats have long been suggested as a major source of phenotypic variation in evolution [46]. This hypothesis was constructed based on the observation that: (1) amino acid tandem repeats are abundant and frequently conserved in the coding sequence of genes involved in development; (2) repeat length variation, both expansion and contraction, occurs in a locus-specific manner 100,000-times more frequently than point mutations; and (3) transcription, mRNA processing, protein synthesis and morphology are affected by length variation in the amino acid repeats [46]. There are, indeed, some examples that offer support to this hypothesis. For example, within dog breeds, there is a clear association between the contraction of glutamine repeats in the *Alx-4* gene and the development of bilateral polydactyly that characterizes the Great Pyrenees breed [46]. Moreover, a correlation was found between the length of the polyglutamine and polyalanine tracts in the *Runx-2* gene and the facial morphology of different dog breeds [46], showing the potential that amino acid repeat variability may assume in the diversification of phenotypes.

Polyalanine repeats are types of amino acid repeats characterized by tandem repeated alanine residues [44]. These regions can modulate protein function through various mechanisms, such as enabling or increasing a transcriptional factor capacity to act as a repressor, modifying the interactome of a protein or acting as spacers, merely providing a structural component that enhances the performance of its functional domains [40,47,48,49]. Interestingly, alterations in the alanine tracts of particular Hox proteins have been proven to impact limb development, to a greater or lesser extent [50,51]. For instance, deletion of one of the HoxD13 alanine polymers in mouse caused fusion of the forelimb sesamoid bones [51,52]. Moreover, increased length of HOXD13 polyalanine repeats has been associated with several cases of synpolydactylism [51,53]. 

HoxA11 and HoxA13 are among the transcriptional factors with crucial function during limb development, where polyalanine repeats have been identified [40,41]. For the human HOXA13, in particular, clear associations were established between the expansion of particular polyalanine tracts (I, III and V) and the development of autopod malformations associated with the human hand-foot-genital syndrome (HFGS) [54,55]. Similar phenotypes were identified in mutant mice with an expanded polyalanine tract [56]. 

**Figure 2:**
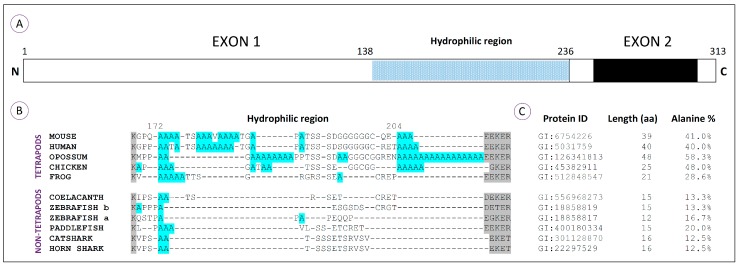
Structure and alanine content of the HoxA11 proteins from vertebrates. (**A**) Schematic representation of the HoxA11 protein, which has 313 aa (amino acid) based on mouse [41,57]. Exons 1 and 2 are represented by white rectangles; the homeobox is in black; and N and C terminal ends are indicated. The variable overall hydrophilic region (98 aa) identified in the N-terminal region is represented by a dotted bar; (**B**) Amino acid sequence alignment of the presumptive hydrophilic region of HoxA11 proteins in vertebrates: mouse (*Mus musculus*), human (*Homo sapiens*), opossum (*Monodelphis domestica*), chicken (*Gallus gallus*), frog (*Xenopus tropicalis*), coelacanth (*Latimeria chalumnae*), zebrafish (*Danio rerio*), paddlefish (*Polyodon spathula*), catshark (*Scyliorhinus canicula*) and horn shark (*Heterodontus francisci*). Alignments were performed with Clustal X [58]. Conserved amino acid residues, flanking the region, are shadowed in grey and alanine residues in blue. Note the presence of polyalanine tracts, with more than four alanine residues clustered, exclusively in tetrapod organisms; (**C**) Identification of the HoxA11 proteins used in the alignment (Protein ID) and the corresponding alanine percentage within the presumptive hydrophilic regions. The sequences used to calculate the alanine percentage correspond to blocks located between extremely conserved residues (in grey). The length of the compared blocks is also indicated here (aa). Note a significant increase of these blocks and a higher percentage of alanine residues in tetrapod organisms, particularly evident in amniotes. Within non-tetrapods, the higher percentage of alanine is found in paddlefish.

Comparative analyses using HoxA11 amino acid sequences from vertebrates reveal that this protein is composed of three major domains [41]: Domains I and III correspond to the N- and C-terminal regions respectively; Domain II is encoded by exon 1 and corresponds to a variable and overall hydrophilic region (Figure 2A). Characteristic reconstructions of Domains I and III suggest that the rates of coding sequence evolution have not changed significantly in tetrapods (frog and chick) relative to lobe-finned fish (coelacanth). However accelerated rates of coding sequence evolution were observed for the mammalian and newt lineages and shown to be a gene-specific phenomenon [41]. Interestingly, when the hydrophilic region of the HoxA11 proteins is compared, stretches of more than three consecutive alanine residues are identified exclusively in the tetrapod sequences, together with an increase in the length of this region (Figure 2B). Thus, the molecular evolution of this transcription factor might have involved an increase of the hydrophilic region by the addition of polyalanine tracts. An hypothetical scenario is that the alanine repeats present in the common ancestor of tetrapods may have acted as a “seed” for the expansion of polyalanine stretches that then contributed to an increased plasticity of HoxA11, potentiating novel functionalities in this lineage, ultimately contributing to the developmental events involved in the fin-to-limb transition [41].

Moreover, the overall alanine content within the hydrophilic region is higher in tetrapods than in non-tetrapod organisms. This is especially evident in amniotes, animals that develop a paddle-like hand plate prior to digit differentiation. In fact, the amphibian’s hydrophilic region lacks polyalanine tracts that appear to be conserved in other tetrapod lineages, and coincidently, the formation of their autopods is not preceded by the development of a hand plate [59,60]. Thus, the addition of alanine residues in the hydrophilic region may have led to novel functionalities of the HoxA11 protein that may have contributed to the origin of the hand plate. Alternatively, the lower alanine content found in the hydrophilic region of amphibians, when compared to other tetrapod linages, may reflect their own evolutionary history that seems to have led to a highly derived morphology [61]. 

Interestingly, in non-tetrapod organisms, the higher percentage of alanine residues in the hydrophilic region is found in the paddlefish, which in fact presents a subtle separation of *HoxA11* and *HoxA13* expression domains at early stages of fin development (Figure 1A; [36]). Thus, hypothetically, higher alanine content in the HoxA11 hydrophilic regions may influence the expression pattern of this protein. Evolutionarily, this could have been the process by which *HoxA11* expression patterns evolved, probably contributing to the fin-to-limb transition.

Given that the repressor domains of transcription factors are commonly small, unstructured, hydrophilic regions with high alanine content [62], these observations suggest that expanded alanine stretches might be enhancers of repressor domains that were instrumental in setting tetrapod-specific features, such as the autopods, as proposed by Chiu and colleagues [41]. However, the search of such a repressor function found no evidence in mouse [63]. Nevertheless, there is still grounds for Chiu and colleagues’ hypotheses, given that the studies performed in mouse just revealed results for a partial deletion of the alanine residues outside the homeodomain, and there is no indication of their location. Thus, just a complete deletion of the alanine residues within the hydrophilic region would be informative. Moreover, the alanine stretches have other predictable roles, such as increasing the plasticity of HoxA11 function, modifying its interactome and/or acting as spacers, providing a structural component crucial to the performance of its functional domains, as suggested by several authors with respect to the function of polyalanine tracts [40,47,48,49]. 

Taking together these analyses, we hypothesize that a sequential increase of the alanine content within the HoxA11 hydrophilic regions may have contributed to: (1) the evolution of *HoxA11* expression patterns during fin development; (2) the establishment of zeugopod/autopod borders; and (3) the formation of the hand plate in the common ancestor of amniotes. 

**Figure 3:**
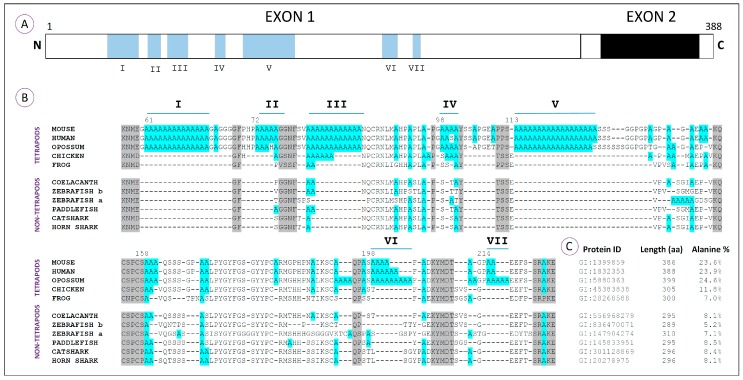
Structure and alanine content of the HoxA13 proteins from vertebrates. (**A**) Schematic representation of the HoxA13 protein, which has 383 aa (amino acid) based on mouse [47,64]. Exons 1 and 2 are represented by white rectangles; the homeobox is in black; and the N and C terminal ends are indicated. Alanine tracts found in the N-terminal region are represented in blue (I–VII); (**B**) Amino acid sequence alignment showing the alanine residues (in blue) detected in the N-terminal region of HoxA13 proteins in vertebrates: mouse (*Mus musculus*), human (*Homo sapiens*), opossum (*Monodelphis domestica*), chicken (*Gallus gallus*), frog (*Xenopus tropicalis*), coelacanth (*Latimeria chalumnae*), zebrafish (*Danio rerio*), paddlefish (*Polyodon spathula*), catshark (*Scyliorhinus canicula*) and horn shark (*Heterodontus francisci*). Note the presence of seven polyalanine tracts (I–VII) exclusively in the mammalian Hoxa13 proteins and conservation of Tract III among amniotes; (**C**) Identification of the HoxA13 proteins used in the alignment (Protein ID) and the corresponding alanine percentage. Note the higher percentage of alanine residues in amniotes, in particular in mammals.

Regarding the HoxA13 proteins, a total of seven polyalanine tracts (I–VII), characterized by a minimum of four clustered alanine residues, has been identified in a variety of vertebrate species at distinct phylogenetic positions (Figure 3A,B; [40,47]). Comparative amino acid analyses reveal that these sequences are characteristic of amniotes, being particularly conserved in mammals (Figure 3B). In fact, Tract III seems to be the only one conserved between mammalian and avian genomes, which suggests that most polyalanine tracts from the HoxA13 proteins arose after the divergence of birds from the lineage that would culminate in the appearance of mammals [40,47]. The incorporation of polyalanine tracts together with flanking segments rich in proline, serine and glycine seems to have increased the N-terminus of mammals approximately 35%, when compared to the fish counterparts [40]. Indeed, while tetrapod HoxA13 proteins have more than 20% alanine content, the zebrafish orthologs have less than 10% (Figure 3C). Within other organisms representing groups that derived prior to the origin of amniotes (frog, coelacanth, zebrafish, paddlefish and sharks), there are alanine residues that align with the polyalanine tracts of amniotes and may represent the primordial elements that gave rise to them. In agreement with this idea, Mortlock and colleagues suggested that the origin of large alanine repeats in mammals might be explained by a dynamic process of recurring replication slippage and point mutation within alanine repeat codons [40]. 

Taking into consideration the potential impact of these polyalanine tracts in the functional plasticity of the proteins, it is possible that their incorporation within the HoxA13 transcription factor may have enabled novel developmental roles contributing to the evolution of the limb structure. However, the absence of such sequences in the HoxA13 of amphibians is intriguing in this scenario. As for HoxA11, we hypothesize that the substantial difference found in the alanine content of the amphibian HoxA13 proteins may explain the lack of a hand plate prior to digit differentiation, which typically characterize the development of amphibian limbs [59]. In order to test this idea, it would be ideal to gain insight into the functional role of Region III, which is conserved in the remaining tetrapod lineages. Several experimental approaches might be relevant to test this hypothesis, including harboring an equivalent region into zebrafish HoxA13, evaluating its capacity to generate a hand plate-like phenotype. Deletions of this particular region in mouse may also be informative to link its functional role to the formation of the hand plate structure. 

**Figure 4:**
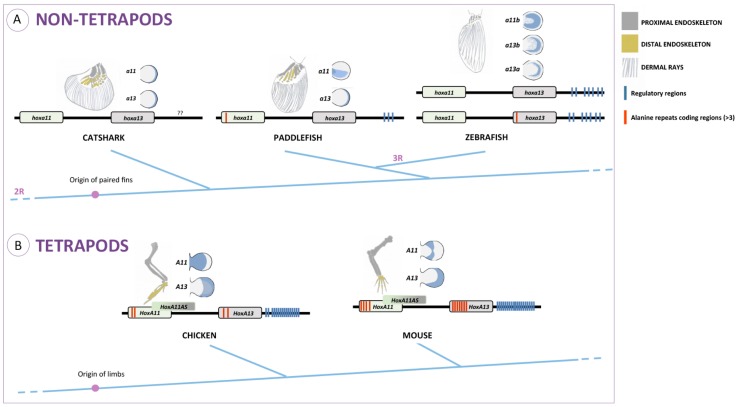
Overview of the morphological, developmental and genomic changes that may have preceded the fin-to-limb transition in vertebrates, comparing non-tetrapod (**A**) and tetrapod (**B**) organisms. Schematic drawing of appendicular skeletons from vertebrates representing distinct phylogenetic positions: basal gnathostomes (catshark); basal actinopterygians (paddlefish); teleosts (zebrafish) and tetrapods (mouse and chicken). 2R and 3R refer to the number of whole-genome duplications that took place prior to the divergence of gnathostomes and teleosts, respectively. Panels adjacent to the appendicular skeletons display *HoxA11* and *HoxA13* expression during appendage development (in blue) based on data from: *Xenopus* [16]; chick [23]; mouse [31]; catshark [35]; paddlefish [36]; zebrafish [17]. The genomic region thought to contain *HoxA11* and *HoxA13* genes in each type of organism is represented under these panels. Note the regions encoding for alanine clusters, with a minimum of three residues (in red) and putative regulatory regions in blue. *HoxA11AS* are the long non-coding *HoxA11* RNAs thought to have an inhibitory effect on *HoxA11* transcription. Question marks in the basal gnathostome condition represent the lack of knowledge of putative regulatory regions operating on 5’ *HoxA* genes. The basal actinopterygian condition was assembled based on data from spotted gar (*Lepisosteus oculatus*). Figure assembly based on [1,40,41,43,57,66,67].

As for HoxA11, a possible outcome of the modifications in the alanine content of HoxA13 proteins may be the enhancement of their role as repressors. An example of such a type of repressive function is well described for HoxA7, in which N-terminal polyalanine repeats are fundamental to enhance its repressive function [65]. This might have enabled this protein to act as a repressor of the *HoxA11* gene during limb development, leading to the establishment of an ancestral zeugopod/autopod boundary and the development of a hand plate. Moreover, dissociation of *HoxA11* and *HoxA13* expression domains may have potentiated the expansion of the skeletogenic process that culminated in the formation of additional endoskeleton elements, such as digits (Figure 4).

Thus, there is in fact grounds to speculate that the incorporation of polyalanine repeats in HoxA11 and HoxA13 proteins may have been an important step for the evolution of limbs. Although a remark has to be made regarding the alanine content of HoxA11 and HoxA13 proteins between fish, amphibians and birds as the comparative alanine content does not support a clear correlation between higher alanine percentage and the presence of an autopod (Figure 2 and Figure 3). Two hypotheses may explain this observation. The first one is that the relatively similar alanine content of amphibians and birds in comparison with fish, but lower in comparison with mammals, results from the different evolutionary paths that those groups experienced [68]. Indeed, some reptiles and amphibians have a remarkable evolutionary history with reference to the evolution of autopod, with squamates having multiple autopod losses, estimated to have occurred at least 25 times independently [69]. Thus, for amphibians, reptiles and birds, a different molecular signature may be correlated with the evolution of the autopod [68]. The second hypothesis is that the contribution of alanines for autopod evolution is not merely quantitative, but also qualitative. However, functional assays are mandatory to make the proof of principle of these hypotheses, as the importance of alanine repeats for limb evolution remains largely unexplored. Taking advantage of the genome editing capacity of the CRISPR-Cas9 technology [70] and the lack of polyalanine repeats in zebrafish [40,41], it would be possible to introduce polyalanine repeats within the zebrafish HoxA11 or HoxA13 and assess how such sequences impact fin development and/or alter the way these transcription factors interact with their downstream targets. The realization of such experiments holds the potential of exploring how homopolymeric amino acid repeats may have impacted the fin-to-limb transition at quantitative and qualitative levels and, on a broader scale, provides evidence of how such repeat expansions may have contributed to the morphological plasticity in vertebrates.

## 3. Non-Coding RNAs and the Fin-to-Limb Transition

In the past decade, the scientific community has discovered that RNA’s biological function is far more complex than the initially proposed role as simple mediators in the DNA-to-protein information flow [71]. Recently, several classes of non-coding RNAs (ncRNAs) with proven biological function have been discovered, such as: long-non-coding RNAs (lncRNAs), microRNAs (miRNAs), Piwi-interacting RNAs (piRNAs), small interfering RNAs (siRNAs), enhancer RNAs (eRNAs) and promoter-associated RNAs (PARs) [72]. Those ncRNAs have various functions, such as suppression of transposon activity and post-transcriptional gene silencing. They may also act as gene transcriptional activators or repressors and be involved in gene epigenetic control [72,73,74]. 

Although exceptions are known, overall, those RNAs seem to have two characteristics in common: a low transcriptional level and a high evolutionary divergence, with their numbers increasing in the genomes along with the species’ evolutionary history [72,75]. For example, a recent study demonstrates that the majority of long-non-coding RNAs in each species are lineage specific [75]. This finding, along with the discovery that ncRNAs are important for proper gene regulation during development in some species [74,76], designates the ncRNAs as possible drivers of species morphological innovation [74]. 

Interestingly, the *HOX* loci produce a considerable repertoire of ncRNAs belonging to two major classes: miRNAs (e.g., *mir-10*, *mir-196*, *mir-99*, *mir-126*) and lncRNAs (e.g., *HOTAIR*, *HOTTIP*, *Mistral*, *HOTATRM1*) [77]. Those ncRNAs have been implicated in the regulation of *HOX* gene expression at the transcriptional, post-transcriptional and translational level [77], and a particularly interesting example of the contribution of such RNA species in the modulation of morphological design comes from mir-196, a well-conserved miRNA in vertebrates [76,78]. McGlinn and colleagues reported that knocking down *mir-196* expression in chicken embryos caused a morphologic alteration of the last cervical vertebrae into a thoracic-like identity [76]. The phenotypic alteration seems to result partially from an expansion of the *Hoxb8* expression domain, caused by the reduction of *mir-196* transcripts [76]. This example shows how relevant miRNAs, and on a broader scale the ncRNAs, are for the regulation of important patterning genes during development [76], with possible evolutionary implications in the establishment of specific morphological traits.

In addition, these ncRNAs may also participate in the epigenetic control of transcription. The lncRNAs HOTAIR was the first one to be discovered to play that role, alerting the importance of RNAs as controllers of the transcriptional activity that may have been highly influential in the determination of the cell fate and, ultimately, in the evolution of animal morphology [79,80]. However, the versatility of functions that these lncRNAs perform during development and disease remains largely unknown. Distinct sources of information (such as conservation, coding potential patterns and anatomical properties) are currently being used to identify the main lncRNA gene families [80]. Regarding the anatomical properties, lncRNAs have been grouped, for example, as: (1) antisense lncRNAs that overlap protein-coding genes; (2) intronic lncRNAs that are encoded within introns of protein-coding genes; (3) divergent lncRNAs encoded in the opposite direction of a protein coding gene, but sharing its promoter; (4) intergenic lncRNAs that are encoded within the intergenic space between protein-coding loci (lincRNAs). 

Among the Hox genes found to be involved in limb development, the *HoxA11* locus stands out as capable of generating several antisense lncRNAs (*HoxA11AS*) in a variety of tetrapods [42,57,74,81] that were found to be polyadenylated and alternately processed [42,57]. Moreover, comparison of these antisense *HoxA11* transcripts revealed tissue-specific differences, as well as aspects of RNA processing conserved during evolution, which strongly suggest that the antisense RNAs from the *Hoxa 11* locus are functionally significant [42].

Two of these *HoxA11AS* have overlapping complementary sequences with the first exon of HoxA11 (ENST00000520360.4 and ENST00000522863.1; Figure 4). In contrast, others seem to have sequence complementary with the enhancer/promoter region of *HoxA11* and appear to lack sequence conservation between fish and mammals (e.g., ENST00000522674.1). In order to evaluate the antisense transcript expression of *HoxA11AS*, Hsieh-Li and colleagues performed *in situ* hybridizations using sense and antisense probes designed for the first exon of *HoxA11* [57]. Surprisingly, they found complementary expression patterns of these probes in mouse embryonic limbs. At E9.5, the *HoxA11* gene is transcribed throughout the distal limb bud region and then gets progressively proximally restricted, up to the point where the *HoxA11* gene is only expressed in the prospective zeugopod region [42,57]. This progressive distal repression of the *HoxA11* gene coincides with the appearance of the representatives of *HoxA11AS* transcripts that begin to be expressed in the most distal limb bud region at E10.5. This antisense expression domain then expands proximally, occupying the prospective autopod region by E11.5 [42,57]. The complementary expression patterns found between the sense and antisense probes designed against the *HoxA11* first exon suggest that production of *HoxA11AS* transcripts during limb development may conduct expression inhibition of *HoxA11* [42,57].

Cross species alignments using the “Comparative Genomic” tool from Ensemble (release 80, May 2015, [82]) suggest that the putative *HoxA11AS* encoding regions have been subjected to a different selective pressure during vertebrates’ evolution. Indeed, the regions that overlap with the complementary sequence of *HoxA11*, in the first exon, seem to be significantly conserved among tetrapods. However, another *HoxA11AS* encoding region, which seems to be complementary to the *HoxA11* enhancer/promoter region (ENST00000522674.1), is apparently less conserved [74]. Within mammals, this particular *HoxA11* antisense is detectable in eutherians and is expressed during embryonic development, but also in the endometrium during pregnancy [74]. The potential role of this *HoxA11AS* in mammal reproduction may justify its generalized presence within eutherians’ genomes. In non-mammal organisms, such as in the frog, the degree of sequence conservation drops to less than 50% [74]. Thus, this amphibian lncRNA, if functional, might be exclusively associated with the regulation of limb development and, as such, under lower selective pressure. Moreover, cross species alignment also revealed an absence of significant similarity between human *HOXA11AS* gene and the syntenic zebrafish, stickleback and coelacanth regions. Moreover, when Bartel and colleagues characterized the lncRNAs during zebrafish development using chromatin marks, poly(A)-site mapping and RNA-Seq data, they did not find any potential homologues of *HOXA11AS*, either by sequence conservation or genomic location [83]. This might be due to the striking lack of conservation of the teleost HoxA intergenic regions, when compared to the ones from other gnathostome lineages [84]. In fact, teleosts have duplicated and more compact HoxA clusters, with shorter intergenic regions [84], which may suggest that they lack most of the genomic region able to transcribe *HoxA11AS*. It remains to be uncovered if fish species that diverge prior to teleost radiation and where the length of the HoxA intergenic regions more closely resemble the ones from the tetrapods have the capacity to produce these lncRNAs (e.g., shark, paddlefish). 

Given the possible inhibitory effect of *HoxA11AS* on *HoxA11* and what seems to be a tetrapod-specific characteristic of *HoxA11AS,* we present the hypothesis that the evolutionary origin of *HoxA11AS* was one of the molecular events that might have contributed to the evolution of the fin/limb morphology. According to this hypothesis, the appearance of *HoxA11AS* expression in the distal limb region inhibits *HoxA11* expression in that limb domain, thus uncoupling *HoxA11* and *HoxA13* limb expression domains and enabling the definition of a distal *HoxA13* single positive autopod domain. The apparent lack of *HoxA11AS* homologues in the zebrafish, according to our hypothesis, may help to explain why the expression domains of *HoxA11/HoxA13* do not split during fin development. In contrast, the transient separation of these domains detected during shark and paddlefish fin development [26,35,36] suggests that these organisms may produce *HoxA11AS*. However, these *HoxA11AS* are probably transcribed in the antisense direction within their own *HoxA11* loci; therefore, they may not be conserved among vertebrates. Taken as inspiration the work from Hsieh-Li and colleagues [57], it would be interesting to analyze the expression pattern of sense and antisense *HoxA11* transcripts in these organisms. 

An important step to gain insight into our preferential hypothesis, which relates the origin of *HoxA11AS* with the evolution of vertebrate fins, is to functionally characterize these transcripts to better understand their biological relevance to limb development. To achieve this goal, a possible strategy would be to take advantage of the chick model, which seems to have conserved *HoxA11AS*, and induce its overexpression, knockdown and knockout *in ovo*, following the strategy used by McGlinn and colleagues [76]. This would allow testing the impact of *HoxA11AS* in the definition of the *HoxA11* expression domain during the formation of limbs. Another important step to explore our hypothesis is to assess the evolutionary importance of these lncRNAs during vertebrate fin/limb development. To this end, an innovative strategy would be the knock-in of *HoxA11AS* in zebrafish and the characterization of its impact during fin development, assuming that the capacity to metabolize these lncRNAs was assembled prior to the divergence of tetrapods.

## 4. *Cis*-Regulation of 5’ *HoxA* Genes and the Fin-to-Limb Transition

According to the previously-presented hypothesis, the definition of the autopod domain is associated with the proximal restriction of *HoxA11* expression during the development of appendicular structures in stem groups of tetrapods [26]. However, the autopod morphology, regarding digit size and number, acquired modifications during evolution probably due to an increase in the transcription of 5’ *HoxA* and *HoxD* genes [8,85,86]. Thus, recruitment of novel non-coding regulatory elements, which are able to modulate the transcription of these genes, seems to have been a crucial step for the evolution of the autopod [85,86]. The enhancer elements that govern the expression of 5’ *HoxD* have been characterized during the development of mouse limbs [85,86]. These reports highlighted that a particular combination of regulatory elements is required for the specification of the appropriate time, dosage and pattern of 5’ *HoxD* gene expression. 

Regarding the *HoxA11* and *HoxA13* genes, despite their crucial role for the correct development and proximo-distal patterning of the limbs, information on their *cis*-regulatory network is still scarce, although the research teams led by Innis [47], Kmita [66], Gómez-Skarmeta and Shubin [43] have shed some light into the enhancers that drive *HoxA13* expression in the developing limbs and their possible impact in the fin-to-limb transition. From the works published to date on mice, the enhancer network that regulates distal *HoxA13* expression in the developing limbs is comprised of at least nineteen different elements located upstream of, and excluding, the *HoxA* cluster (Figure 4) [66]. Some of those enhancers are present within genes located upstream of the *HoxA* cluster, namely the *Hibadh*, *Tax1bp1* and *Jazf1* genes [66]. A curious observation is that the enhancers driving *HoxA13* distal expression in mouse limb form several sub-megabase topological domains (sub-TADs) that contact each other in a fashion that, in some cases, seems to be independent of the enhancer’s transcriptional activity. This suggests that the sub-TADs organization is governed by a developmental structure/function interplay instead of a mere clustering of active enhancers [66]. 

When the conservation of the mouse *HoxA13* enhancer network was assayed, using sequence homology and ATAC-Seq (assay for transposase-accessible chromatin using sequencing) in zebrafish and gar, only the gar genome showed conservation of three (e10, e13 and e16) of the 19 enhancers that regulate *HoxA13* transcription in mouse limb, and at least the gar e16 region induces, in zebrafish and mouse transgene reporter assays, an expression pattern that is very similar to the one driven by mouse e16 [43]. Taking into account that lack of sequence conservation is not necessarily sufficient to exclude the presence of enhancers in fish, these data nevertheless suggest two possible circumstances. Firstly, the zebrafish *hoxa13a* and *hoxa13b* enhancer network, if present, represents a derived state, as no sequence homology was detected between zebrafish, gar and mouse *HoxA* enhancers [43]. This observation may result from extensive remodeling of *HoxA* regulatory elements caused by the occurrence of a third whole genome duplication in teleosts followed by gene sub-functionalization, thus leading to the inability of the zebrafish *hoxa13* paralogs to respond to a “distal limb program” during development [43]. Secondly, the enhancer network that regulates *HoxA13’*s late phase expression suffered extensive elaboration/remodeling over the course of the vertebrates’ evolution, although some of its elements might have already been present in the last common ancestor of bony vertebrates [43]. The extensive elaboration of *HoxA13* enhancer elements throughout vertebrate evolution suggests that the fish *HoxA13* transcriptional level during fin development is too low to elicit an adequate “distal limb identity”, thus suggesting that *HoxA13* gene dosage increase was a requirement for the fin-to-limb transition. This hypothesis finds support in the work published, in 1997, by Zákány and colleagues [8], where by analyzing a set of distal *HoxA* and *HoxD* compound mutants, this team found that overall autopod morphology, and in particular digit number and length, greatly varies in response to *Hox* dosage [8] and to a minor extent to the qualitative nature of the expressed HoxA and HoxD proteins (*Hox* code) [85]. Moreover, overexpression of zebrafish *hoxd13a* was shown to cause changes in the morphogenesis of the fins towards a limb-like identity [22]. Testing out the hypothesis that an increase of *HoxA13* dosage was an evolutionary requirement for the fin-to-limb transition could be easily achieved in zebrafish following a strategy previously described by Freitas and colleagues [22]. Performing such a proof-of-principle study not only upholds the potential to disclose how increasing levels of HoxA13 proteins may have contributed to the morphological transition between fish fins and tetrapod limbs, but may also shed light on how regulation and function of *Hox13* paralogs genes may have set the grounds for autopod evolution.

## 5. Conclusions and Future Directions

In this review, we attempted to survey the evolution of HoxA11 and HoxA13 transcription factors and their regulatory control in vertebrates. Evaluating the current state of the art and performing complementary analyses, using information currently available for distinct vertebrate lineages, we support the hypotheses implicating changes in the alanine content, capacity to generate long-non-coding RNAs and incorporation of a novel *cis*-regulatory region in the developmental mechanisms involved in the fin-to-limb transition in vertebrates (Figure 4). 

Conserved polyalanine tracts with a minimum of four residues are common within the HoxA11 and HoxA13 proteins of tetrapods (Figure 2, Figure 3 and Figure 4). However, these types of sequences are undetectable in the orthologs of fish. In the HoxA11 proteins, these enriched alanine domains co-localize with the hydrophilic region, being higher the percentages of alanines detectable in amniotes. Among the fish analyzed, the hydrophilic region of paddlefish seems to be the one that has a higher content of alanines. These observations point us to two hypotheses: (1) an increased alanine content within HoxA11 proteins may have impacted the formation of specific amniote limb structures during evolution, such as the hand plates that precede digit differentiation; (2) incorporation of extra alanine residues into the hydrophilic regions of HoxA11 protein probably started prior to the tetrapod divergence, in the common ancestor of actinopterygians and sarcopterygians. In the HoxA13 proteins, most polyalanine tracts (I–VII) are conserved in mammals (Figure 3 and Figure 4). However, the polyalanine Tract III seems to be present in the chick HoxA13 proteins, which may suggest a particular role of these tracts in the formation of amniote-specific limb structures, the hand plates. 

Taking into consideration the functional potential found for this type of amino acid repeats [40,47,49], their integration into the HoxA11 and Hoxa13 proteins during evolution might have modified the way they interact with their downstream targets, contributing to the phenotypic variation found in the evolutionary transition from fish fins to tetrapod limbs. For example, the polyalanine repeats found in the HoxA11 and HoxA13 proteins might have enhanced their repressor role upon other molecules, helping to establish novel domains of cell identity, leading to the differentiation of the hand plate in amniotes. The repressor potential of HoxA13 in particular might have inhibited the expression of *HoxA11* in the most distal mesenchyme of the appendages, generating a novel field of cell identity, which might have potentiated the formation of additional skeletal elements. However, the proof of principle of these hypotheses is lacking. Here, we suggest, as a future working perspective, the use of CRISPR-Cas9 technology [70] to harbor polyalanine repeats within the zebrafish *HoxA11* or *HoxA13* in order to evaluate the phenotypic consequences of such sequences during appendage development. These experiments would certainly add to our knowledge of how changes in the alanine content of transcription factors impacted the diversification of vertebrate structures.

In addition, we show here that the capacity to generate *HoxA11AS*, which are long-non-coding RNAs, seems to be exclusive to tetrapods (Figure 4) [42,57,74,81]. Moreover the expression of particular *HoxA11* antisense RNAs was found to be complementary to *HoxA11*, suggesting that while *HoxA11* is progressively repressed distally, *HoxA11AS* expression expands proximally. This observation points to a repressor role of *HoxA11AS* on *HoxA11* that might have contributed to split the *HoxA11/HoxA13* expression domains, potentiating the formation of the zeugopod and the autopod. Given that during catshark and paddlefish fin development, the expression domains of *hoxa11* and *hoxa13* also split transiently into two non-overlapping territories, it would be important to search for *HoxA11AS* homologues in these organisms and evaluate the potential repressor role on *HoxA11*. These ideas also lack functional validation, and a first step would be to gain more insight into the expression of HoxA11 antisense in fish representing distinct phylogenetic positions. Moreover, it would be important to better characterize the role of *HoxA11AS* during tetrapod limb development, to test their developmental and evolutionary potential harboring of *HoxA11AS* in zebrafish and to characterize the impact during appendage development.

Finally, we suggest that the transcriptional levels of *HoxA11* and *HoxA13* might have also influenced appendage evolution, as has been proposed for *HoxD13* [22]. Indeed, the autopod morphology in mouse, in particular digit number and length, is influenced by the *Hox* dosage [8], which often depends on the enhancer capacity of *cis-*regulatory regions within their genomic landscape [87]. Marked differences in the putative regulatory regions of the 5’ *HoxA* genes were identified in fish species representing distinct phylogenetic positions, such as the spotted gar and the zebrafish (Figure 4). Thus, *cis*-regulatory changes may have interfered with the differential fin morphology of distinct fish groups (Figure 4). Within tetrapods, subtle changes were also identified in the putative regulatory regions of these genes that may have contributed to the distinct autopod anatomy found in mammals and birds, for example [43]. Additionally, fish *versus* tetrapod comparisons suggest a significant increase of *cis*-regulatory modules in tetrapods [43], a phenomena that might have been crucial to achieve the appropriate levels of Hox expression required for the origin of the autopod. To further explore the consequences of increased levels of 5’ HoxA proteins and the addition of novel regulatory modules for the developmental processes resulting in the evolution of limbs, we emphasize the advantages of the zebrafish model to perform the appropriate proof of principle studies due to its current technical repertoire, allowing a variety of functional assays [22].
